# Soil Temperature Triggers the Onset of Photosynthesis in Korean Pine

**DOI:** 10.1371/journal.pone.0065401

**Published:** 2013-06-03

**Authors:** Jiabing Wu, Dexin Guan, Fenhui Yuan, Anzhi Wang, Changjie Jin

**Affiliations:** State Key Laboratory of Forest and Soil Ecology, Institute of Applied Ecology, Chinese Academy of Sciences, Shenyang, P.R. China; University of Illinois, United States of America

## Abstract

In forest ecosystems, the onset of spring photosynthesis may have an important influence on the annual carbon balance. However, triggers for the onset of photosynthesis have yet to be clearly identified, especially for temperate evergreen conifers. The effects of climatic factors on recovery of photosynthetic capacity in a Korean pine forest were investigated in the field. No photosynthesis was detectable when the soil temperature was below 0°C even if the air temperature was far beyond 15°C. The onset of photosynthesis and sap flow was coincident with the time of soil thawing. The rates of recovery of photosynthetic capacity highly fluctuated with air temperature after onset of photosynthesis, and intermittent frost events remarkably inhibited the photosynthetic capacity of the needles. The results suggest that earlier soil thawing is more important than air temperature increases in triggering the onset of photosynthesis in Korean pine in temperate zones under global warming scenarios.

## Introduction

In temperate forests, the onset of spring photosynthesis may have an important influence on the carbon assimilation of trees [1], [2] and hence influence the carbon balance of forest ecosystems [3]. The onset of photosynthesis is a crucial process in plant phenology. Accurate evaluation of the starting date of photosynthesis and its connections with future climatic factors are essential for understanding and modeling the ecosystem carbon balance. For example, enhanced warming at high northern latitudes has been reported to result in the earlier onset of plant photosynthesis and therefore an increase in forest productivity [4]–[5].

The photosynthetic capacity of deciduous trees in boreal and temperate forests varies considerably with the annual cycle of presence and absence of foliage. Therefore, for deciduous trees, the onset of photosynthesis in spring is linked to the easily observable growth of leaves. Several studies report that air temperature triggers the recovery of boreal and temperate deciduous forest photosynthesis in spring (e.g., [6]–[7]), and the effects of air temperature on the onset of photosynthesis have been well modeled [7]. Accordingly, an earlier onset of photosynthesis in spring for these forests is reported following global warming [8]–[9].

Evergreen coniferous trees growing in boreal and temperate zones have a clear annual cycle of growth and dormancy period, considering that the photosynthetic capacity of the needles is generally inhibited during the wintertime by low air temperature [10]. However, variations in the timing of onset of photosynthesis as well as its determining factors, are not easily detectable. Tanja et al. (2003) [11] found that air temperature is the main trigger of the onset of plant photosynthesis at five evergreen boreal coniferous field stations. Sevanto et al. (2006) [12] found that the photosynthesis of *Pinus sylvestris* begins when the air temperature reaches 3°C to 4°C, even though the soil remains frozen, and argued that water for needle photosynthesis is possibly available from stored stem water. However, several temperature treatment experiments and field investigations on evergreen conifers have also shown that the onset of photosynthesis cannot be observed even after several days of exposure to optimal air temperature when the soil is frozen [8], [13]–[14]. Some studies have reported that soil temperature is the primary control of boreal conifer photosynthesis and related this effect to the inhibition of water uptake from frozen soils (e.g., [15], [16]). A literature overview shows that there are extensive studies on the seasonal development of tree photosynthesis in temperate forests. However, further studies are still need to improve the understanding of the factors that trigger the onset of photosynthetic recovery for temperate evergreen coniferous trees.

The global temperature is expected to rise in future decades, and warming has been predicted to be especially pronounced during spring in regions located at mid-and high latitudes [17]. To further our knowledge of the effects of climate and climate change on plant phenology, existing worldwide CO_2_ flux measurements from the micrometeorological tower sites provide a valuable resource to investigate the effects of climate on photosynthetic phenology across biome and climate zones. Using long-term measurements of net ecosystem carbon exchange from an eddy covariance flux tower, and concurrent measurements of needle photosynthesis, sap flow in trees, and associated meteorological variables, we attempt to determine the environmental factors initiating the onset of photosynthesis for Korean pine in the temperate forest of Northeast China. In particular, we evaluate the importance of soil temperature versus air temperature in determining the onset of photosynthesis in temperate evergreen coniferous trees.

## Methods

### Ethics Statement

The field studies were conducted at the Changbai Mountains National Nature Reserve, in northeastern China. It is a practice base for the researchers of the Chinese Academy of Sciences. Thus we could conduct experiments there without specific permits. The experiments conducted in this study do not involve or impact endangered or protected species.

### Observation Sites and Climatic Background

The experimental site is located within the National Natural Conservation Park of Changbai Mountains, northeast of China (42° 24′ 10″ N, 128° 05′ 46″ E, with an elevation of 740 m). The study area lies in a monsoon-influenced, temperate continental climate zone. Its annual mean temperature is 3.6°C, and its annual mean precipitation is 690 mm. The site is vegetated with virgin old-growth Korean pine (*Pinus koraiensis*) as dominant species and interspersed with tuan linden (*Tilia amurensis*), Mono maple (*Acer mono*) and larch (*Olgensis* var.). The mean canopy height is about 27 m and its maximum leaf area index is 6.0 [18].

### Observations and Data Processing

A flux tower was located in the Korean pine forest in Changbai Mountains. Fluxes of CO_2_ and H_2_O were measured using eddy covariance methodology at a 60.0 m high micrometeorological tower. Air temperatures and three-dimensional wind speeds were measured with a three-dimensional sonic anemometer (CSAT3, Campbell Sci., Inc., USA). A fast-response, open-path infrared gas analyzer (LI-7500, LI-Cor Inc., USA) was used to measure CO_2_ and H_2_O concentrations. Analog signals from the sonic anemometer and the infrared gas analyzer were sampled by a data logger (CR3000, Campbell Sci., Inc., USA) at 10 Hz.

Fluxes of CO_2_ were calculated at 30 min intervals. Linear trends in the 10 Hz raw data of air temperature and CO_2_ concentrations were removed. The wind components measured by the sonic anemometer were rotated so that the mean transverse velocity and the mean vertical velocity were reduced to zero for each 30 min interval [19]. Transfer functions were applied to correct the high-frequency CO_2_ flux losses caused by the sensor separation, and the frequency response of the data acquisition system [20]. CO_2_ fluxes were corrected for density fluctuations arising from air temperature and humidity [21]. Finally, the flux data were screened using an integral turbulence characteristics test following Foken and Wichura (1996) [22], which removed data points associated with disturbed and underdeveloped turbulence. The percentages of the remaining flux data in the study period for 2006, 2007, 2008, and 2009 were 57.4%, 63.8%, 57.0%, and 67.1%, respectively. Data gaps were filled by using standardized methods: linear interpolation for small gaps shorter than 2.0 hr and mean diurnal variation method for longer gaps [23].

CO_2_ flux measurements spanning from April to May (DOY 91 to DOY 151) in the years 2006 to 2009 were used. These periods fully covered the stages of soil thawing, onset of needle photosynthesis, and full development of foliage photosynthesis. The onset of photosynthesis in trees is actually a gradual process but we treated it as an on-off phenomenon in this study for simplicity. In the current study, we defined the onset of photosynthesis as the point when the three-day moving average of integrated net ecosystem carbon exchange during daytime (*NEE*_d_) first falls below zero. Daytime was defined as the period in which net radiation is greater than 10 W·m^−2^.

Soil temperatures were measured using multilevel thermocouple probes, with two sets of sensors (105T, Campbell Sci., Inc., USA) placed at 5, 10, 20, and 50 cm below the soil surface under the canopy of pine trees. Air temperature was measured with one set of HMP45C probes (Vaisala, Helsinki, Finland) placed at 2, 16, 22, 26, 32, and 60 m height of the flux tower. Net radiation was measured with CNR-1 net radiometer (Kipp & Zonen, Delft, Netherlands) at 40 m height. These ancillary temperature measurements responded to frequencies up to 0.5 Hz. Six Granier-type thermal dissipation probes (TDP30, Rainroot Scientific Limited, Beijing, China) were used to measure the sap flux density of tree stems. Two sensors were inserted into each of the three pine stems at 1.5 m height. One sensor was located on the eastern side and another was placed on the western side of the stem.

Light response curves were performed using a portable photosynthetic analyzer (LI-6400; LI-Cor Inc., USA) equipped with a standard 3 cm×2 cm leaf chamber and a red/blue light source (6400–02B). Three pine trees were chosen and photosynthesis measurements on each tree were carried out daily for 15 consecutive days during the soil thawing period. Measurements were performed on branches (about 2 cm in stem diameter) that were cut from the mid-portion of the canopy and were immediately placed in water. Pine needles were allowed to adapt to each light level for 3 minutes before each point was recorded.

## Results

### Environmental Information

**Figure 1:**
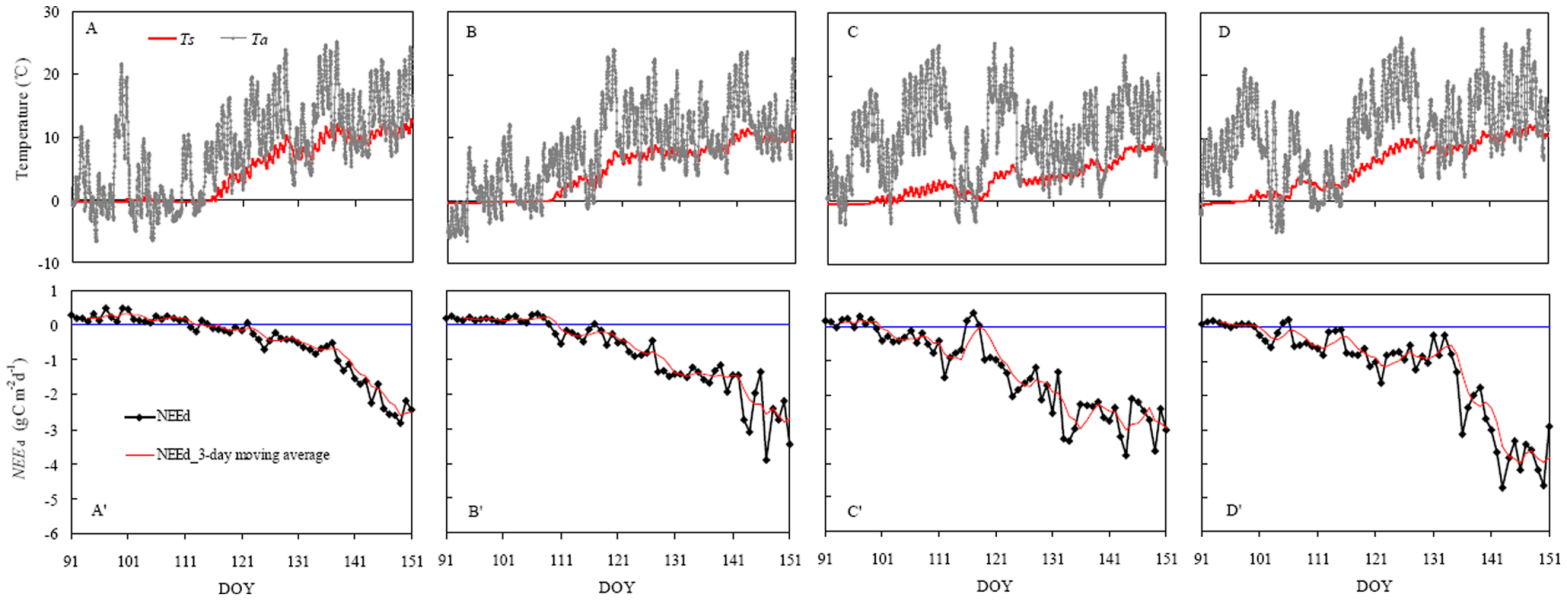
Seasonal courses in three-day moving average of *NEE*_d_ (daily integrated net ecosystem carbon exchange), the 26-m height air temperature and 5-cm depth soil temperature at the Changbai Mountains Korean pine forest from DOY 91 to DOY 151 (1^st^ of April to 31^st^ of May) in year of 2006 (A–A’), 2007 (B–B’), 2008 (C–C’) and 2009 (D–D’).

Meteorological information on the Korean pine forest is presented in Table 1. In general, remarkable annual variations in the monthly mean values of temperature and precipitation in April and May were observed, especially in April of 2006, which showed the lowest air temperature (Fig. 1A) and precipitation. Although statistically insignificant, possibly due to the limited observed time range, a trend of warmer springs (0.97°C/yr, *r* ^2^ = 0.84, p<0.1) was observed in the study area, as suggested by the linear fitting of the mean air temperature during April and May over the four-year period. Hence, a trend of earlier end of the soil thawing period (from DOY 117 to DOY 98) was observed, as indicated by measured soil temperatures at a depth of 5 cm.

**Table 1:** The basic information on meteorological factors, monthly cumulative net ecosystem carbon exchange rates (*NEE*_d_) and the dates of onset of photosynthesis investigated from Changbai Mountains forest site during study period.

Year	Month	Air temperature (°C)	Precipitation (mm)	Cumulative *NEE*_d_ (gC·m^−2^)	Date of soil unfrozen (DOY)	Onset of photosynthesis (DOY)
2006	April	3.2	23	5.6	117	117
	May	13.1	50.6	−36	–	–
2007	April	3.9	33.9	1.1	109	110
	May	11.9	93.3	−46.4	–	–
2008	April	9.4	33.0	−3.2	98	99
	May	10.6	72.5	−75.2	–	–
2009	April	6.8	36.5	−9.9	100	100
	May	14.6	46.7	−63.9	–	–

Details on the variation in temperature are shown in Fig. 1. Air temperature varied widely during spring photosynthesis recovery period. The monthly air temperature in April over four years of observation averaged 5.8°C. The minimum air temperature, which was observed in 2006, was 3.2°C, and the maximum air temperature, which was observed in 2008, was 9.4°C. Earlier soil thawing corresponding to warmer springs was demonstrated by the annual variation in soil temperature. During the observation period, 26 m height air temperature showed significant variations from –6.5°C to 20.8°C before the onset of photosynthesis, while 5 cm depth soil temperatures remained constant at around 0°C (isothermal stage), and the period of isothermal stage generally lasted for around three weeks each year. The earliest start of soil thawing at 5 cm depth was observed in early April 2008 (DOY 98), and the latest start of soil thawing was observed in April 2006 (DOY 117). Soil temperatures rose rapidly after the end of soil thawing, reaching a maximum of 13.0°C by the end of May in 2006.

### Patterns of Photosynthesis and its Controls

The timing of annual onset of photosynthesis ranged from DOY 99 to DOY 117, with an average starting date of DOY 107. The earliest start of photosynthesis appeared in April 2008 (Tab. 1), coinciding with the highest monthly mean air temperature. The latest signs of photosynthesis appeared in April 2006, coinciding with the appearance of the lowest monthly mean air temperature. However, data analysis suggests that air temperature is not the main trigger of the onset of tree photosynthesis, as observed from seasonal evolutions of air and soil temperature and synchronous variations in the daytime net ecosystem carbon exchange (Fig. 1). The onset of photosynthesis coincided with the end of soil thawing, and the maximum lag of onset of photosynthesis related to the end of soil thawing was generally within one day. After onset of photosynthesis, photosynthetic rates obviously fluctuated with daily and seasonal evolutions of air temperature throughout the study period.

**Figure 2:**
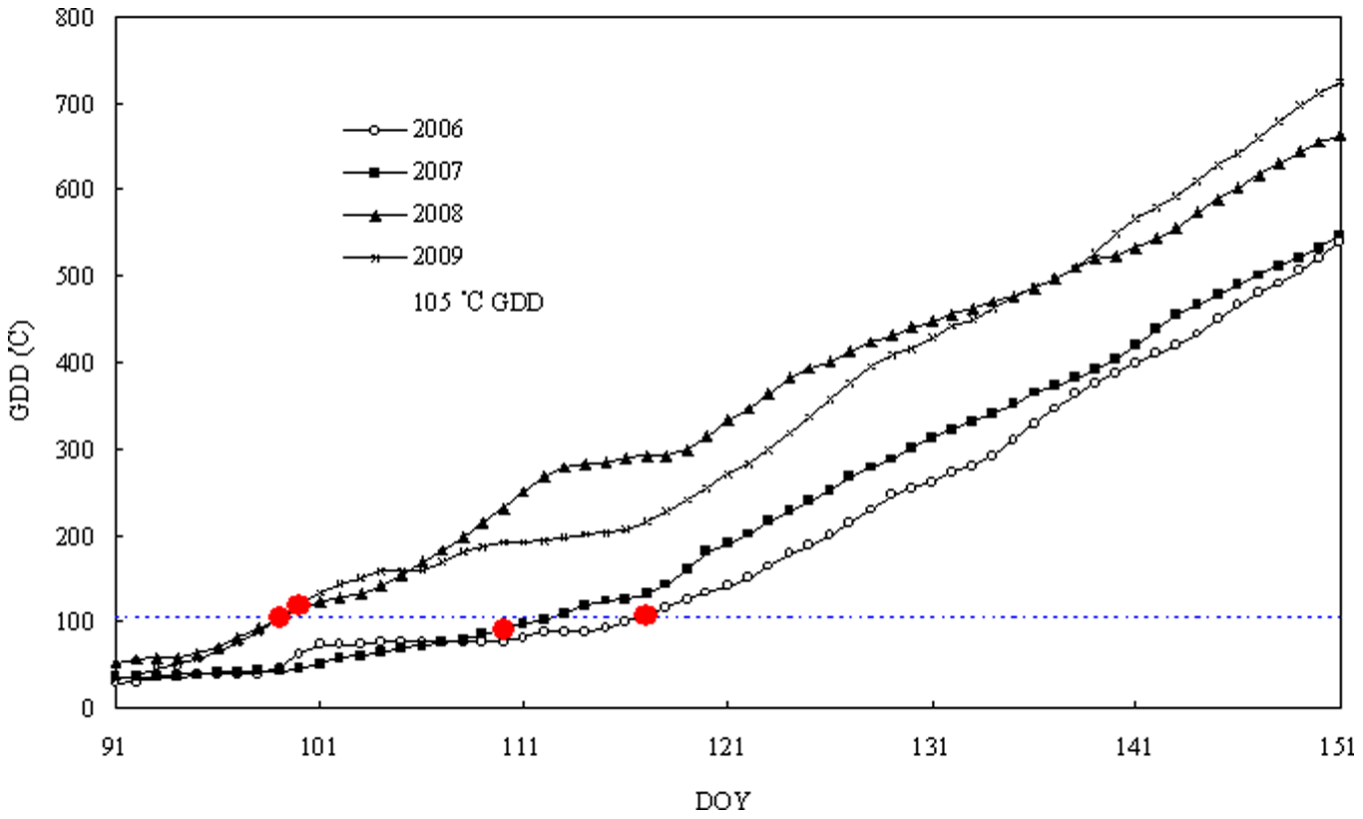
Onset of Korean pine photosynthesis as denoted by accumulated growing degree days (GDD) measured at canopy height (26 m). Horizontal lines mark the 105°C isotherm of the GDD threshold. The circles denote the timing of onset of photosynthesis in each year.

Further data analysis suggests the existence of a threshold value of accumulated growing degree days (GDD, which is defined as a cumulative sum of daily mean air temperatures above 0°C from the beginning of the soil isothermal stage period) related to the timing of onset of photosynthesis. As observed from Fig. 2, photosynthesis began when the GDD had accumulated to 105°C in all of the observed years. The GDD was significantly higher in 2008 and 2009 than in 2006 and 2007, obviously contributing to the earlier onset of photosynthesis in the latter two years.

**Figure 3:**
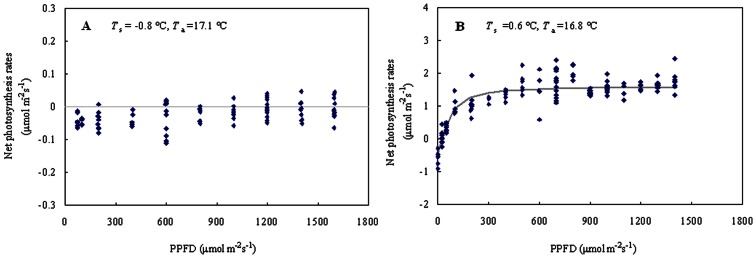
Light response curves for needles of Korean pine measured one day before soil thawing (A) and two days after the soil temperature was above freezing (B). PPFD is photosynthetic photon flux density, *T*_s_ is soil temperature and *T*_a_ is air temperature.

*In situ* measurements of photosynthetic light response curves with a LI-6400 portable photosynthesis system also suggested that the pine needles could not begin photosynthesis when soil is frozen. As can be seen from Fig. 3, after 20 min of adaptation to saturated light, photosynthetic carbon uptake was not detected in one-year-old pine needles even though the air temperature was far beyond 15°C while soil temperature *T*_s_ was below 0°C. Comparably, photosynthetic activity was detected immediately at the end of the isothermal period (*T*_s_ reached 0.6°C).

**Figure 4:**
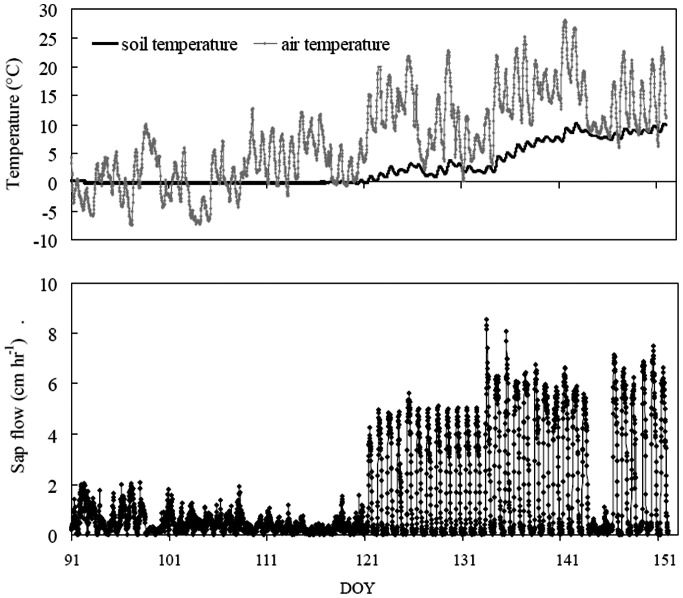
Seasonal evolution of air and soil temperature and sap flow rates measured in Korean pine stems in the early spring of 2010.

The effects of soil thawing on tree photosynthesis were also well reflected by measurements of sap flow. As observed from Fig. 4, early in the spring, when the pine needles were still dormant, air temperatures rose above freezing during the day but dropped back below freezing at night. Sap flow was not initiated in the tree until the soil temperature in the upper rooting zone (0–20 cm depth) soil had reached a certain temperature above 0°C, even when the atmospheric water demand was likely large under high air temperature conditions. Pines with favorable air temperature (up to 13.2°C) did not take up water until the end of the thawing period of the surface soil.

Further analysis suggests that the low air temperature obviously inhibits photosynthetic recovery after onset of photosynthesis in Korean pine. An extreme case of low temperature inhibition was the occurrence of frost. We defined a “frost event” as an average temperature decrease of at least 10°C within 24 hours, and simultaneously the minimum temperature drops down to 0°C or lower. Frost events occurred during recovery of photosynthetic capacity in all of the observed years except in 2006, as shown in Table 2. Generally, frost events caused declines of over 80% in net photosynthetic rates and even caused transitions from net carbon assimilation to net carbon emission in the studied forest in the daytime. Frost-induced inhibition of photosynthesis was more severe in 2008 than in other years because frost events occurred comparatively late in the recovery period. As we can see from Fig. 1C, a continuous three-day net carbon emission was observed from DOY 116 to DOY 118 even though photosynthesis had already fully developed by this time. Recovery of photosynthesis to pre-frost levels was completed during the subsequent two to three days, and the recovery duration was dependent on the severity of frost.

**Table 2:** The inhibitory effects of frost on recovery of photosynthetic capacity in Korean pine in the Changbai Mountains region over the four observation years.

Year	Date of occurrence of frost (DOY)	Time duration of onset of photosynthesis (days)	2 days pre-frost mean *NEE*_d_ (gC·m^−2^ ·d^−1^)	2 days post-frost mean *NEE*_d_ (gC·m^−2^ ·d^−1^)	Recovery duration (days)
2006	–	–	–	–	–
2007	116	6	−0.42	−0.08	2
2008	115	16	−0.72	0.25	3
2009	104	4	−0.49	0.19	2

## Discussion

The timing and rate of recovery of photosynthetic capacity in spring influence the potential carbon balance of boreal and temperate forests. Several approaches have been used to define the seasonal start of ecosystem photosynthesis. Tanja et al. (2003) [11] defined the onset of photosynthesis in five boreal coniferous forests as the date when the half-hourly *NEE* first falls below 20% of the maximum summer *NEE*. Moore et al. (2006) [24] defined the onset of photosynthesis in a temperate bog as the date when the estimated daily photosynthesis (GPP) first becomes greater than zero. In the current study, the onset of photosynthesis in Korean pine forest was defined as a cumulative daytime *NEE* less than zero. This definition avoids the uncertainties in GPP estimations, considering potential errors in estimating daytime ecosystem respiration fluxes because of the light inhibition of leaf respiration [25]. Besides, without consideration of the high nighttime ecosystem respiration of forests with high biomass, *NEE*_d_ can more sensitively reflect the development of plant photosynthesis. It has to been mentioned that for this proposed method, the onset of photosynthesis will be detected only when the daytime photosynthesis will exceed daytime ecosystem respiration, which could lead to biases, depending on the strength of ecosystem respiration in the spring season. However, from another perspective, this method can effectively prevent the misjudgment caused by the disturbance of earlier onset of photosynthesis of early-spring herbs growing on the forest floor. According to the investigation conducted on April 21, 2005 [26], there were 12 herb species (including *Anemone amurensis*, *Adonis amurensis*, *Corydalis repens*, and so on) growing on the floor of this pine forest and their biomass was 588.7 kg·hm^−2^.

Based on global scale correlations between NDVI and environmental factors, Schultz and Halpert (1993) [27] found that onset of spring photosynthesis is mainly influenced by temperature in cold regions, by both precipitation and temperature in temperate regions, and by precipitation in regions where the amplitude of the annual temperature cycle is small. Such responses have been verified by investigations at the ecosystem scale and individual plant level [24]. Overall, the site-based research results are generally consistent with regional scale findings, but each dataset presents special properties. Suni et al. (2003) [9] reported that air temperature triggers the recovery of evergreen boreal forest photosynthesis in spring. Ensminger et al. (2006) [28] also considered that air temperature is responsible for the onset of photosynthesis in spring. However, onset of photosynthesis in plants has been mostly attributed to soil temperature-related triggers. For example, by using eddy covariance technique, Hollinger et al. (1999) [29] found that significant ecosystem carbon uptake of an evergreen coniferous in the Howland Forest site began with the thawing of the soil in early April, and hence concluded that the threshold for the regulation of photosynthetic carbon assimilation is the spring thawing of forest soil. Control experiments on soil temperature also suggest that the timing of onset of photosynthesis is delayed by delayed soil thawing [30] and advanced by earlier soil thawing [31].

In the current study, measurements from the eddy covariance and sap flow systems showed that no photosynthetic activity (Fig. 1) or root water supply for photosynthesis (Fig. 4) was available in Korean pine while the soil was frozen despite the daily mean air temperatures have reached and maintained above 10°C for several weeks. The sap flow measurements suggest that water uptake of roots is restricted when soil is frozen, limiting water flow into the trees and, ultimately, photosynthesis. Whilst photosynthetic measurements of pine needles with the LI-6400 portable photosynthesis system suggested that no photosynthesis occurred while the soil was frozen despite the air maintaining a temperature of over 15°C (Fig. 3). Immediate photosynthetic responses to soil thawing verified that soil temperature is essential for the onset of photosynthesis in temperate coniferous trees. This study case also suggests that once air temperatures permit photosynthesis, the availability of unfrozen soil water triggers the onset of pine photosynthesis. Similarly, Havranek and Tranquillini (1995) and Monson et al. (2005) [32]–[33] found that photosynthesis could not begin recovering until liquid water is available in the soil. Jarvis and Linder (2000) [16] argued that photosynthesis couldn’t commence in evergreen trees until the soil has thawed because the onset of plant photosynthesis must be accompanied by water supply to leaves and it would be impossible for trees to take up water from frozen soil.

Fig. 1 shows that forests can carry out photosynthesis with air temperature at canopy height below 0°C after onset of needle photosynthesis. For example, the observed data on April 22, 2009 showed that the maximum air temperature was –0.42°C, while the daily *NEE*_d_ value was close to, but smaller than zero (Fig. 1D). Substantial reductions but not total inhibited in needles photosynthesis in early spring indicated that subfreezing daytime temperatures had a large impact on gas exchange because soil temperatures decreased slightly or did not change during this period. This finding further demonstrates that the importance of air temperatures on gas exchange after onset of photosynthesis is remarkable. However, from another perspective, it also suggests that it is soil temperature, rather than air temperature, that determines the onset of photosynthesis under natural field conditions. Reversible inhibitions of net photosynthesis have been observed in Scots pine seedlings in the field with temperatures below the freezing point of needles [34]. Monson et al. (2005) have noted that in evergreen pine and spruce forests the spring recovery can be reversible, after a cold spell [33]. However several studies also reported that temperate evergreen coniferous forest maintains photosynthetic carbon assimilation in winter [29], [35]–[36], even in cool-temperature regions with cold continental winters [37]. Currently less is known about the factors trigger the switch on or off of tree photosynthesis in winter or early spring. Our results contribute to the limited literature on the photosynthetic phenology of temperate trees by showing that soil temperature is a primary factor determining the timing of onset of temperate conifer photosynthesis and that the rate of photosynthetic recovery is generally limited by low air temperatures.

One of the most significant findings from this study is that the recovery of photosynthetic capacity can be reversed by a frost event in evergreen coniferous forests. Such events frequently occur at the stage of spring photosynthetic recovery in boreal and temperate forests. Even a short-term frost event during the transitional periods from dormancy to growth in early spring could result in remarkable damage to the pine forests [38]. Recovery of net ecosystem carbon exchange rates of pine forest from frost is completed about two to three days after a temperature increase. Davidson et al. (2004) [39] found that recovery of photosynthesis to pre-frost levels of evergreen eucalyptus trees required around three consecutive frost-free nights and depended on the severity of frost. Both cases of recovery duration for evergreen trees are faster than the recovery of deciduous forests. For example, Oksanen et al. (2005) [40] reported that recovery of photosynthesis of birch forests from frost has not been completed even after 14 days. This finding is an interesting phenomenon worthy of further study in the future. A possible explanation for this different photosynthesis recovery ability is that structural injuries to the foliage of deciduous trees are more severe than that of evergreen trees.

Similar to other studies [41], [42], the timing of onset of photosynthesis is a function of the accumulated temperature sum above a given threshold (Fig. 2), which suggests that the accumulation of heat controls onset of photosynthesis. The GDD is a good predictor of the onset of photosynthesis suggesting that the accumulation of heat controls onset of photosynthetic activity. However, it is not a general principle that plant photosynthetic recovery responses to a threshold GDD, considering that an arbitrary threshold temperature (e.g., 0, 5, and even 10°C) is used for calculating GDD in different studies. In addition, frost can reverse recovery in photosynthetic capacity [43], [44], and, therefore, stochastic frost events can confound the simple relationship between photosynthetic recovery and GDD. In the current study, we chose to calculate GDD based on the beginning of the soil isothermal stage because this time corresponds to the possible availability of liquid water from thawing soil.

The timing of the spring thaw varies between years. In the current study, the difference of 19 days between the earliest (year of 2008) and the latest (year of 2006) soil thaw represents an increase of almost 13% in season length. It is consistent with the statement that the length of the northern hemisphere growing season may be increasing and this may affect carbon uptake of terrestrial ecosystem [45].

### Conclusions

In the current study, the effects of soil and air temperature on the spring recovery of photosynthesis in pine forest were assessed in the field. The onset of photosynthesis in Korean pine was closely related to the end of soil thawing, and the rate of recovery of photosynthetic capacity was largely dependent on air temperature. The pine needles could start photosynthesis immediately after soil water became available for the roots. Intermittent frost events remarkably inhibited recovery of photosynthetic capacity in evergreen conifers during spring; this effect was reversible immediately after the temperature was raised back to former values. The results suggested that soil temperature is more important than air temperature in determining the onset of photosynthesis in Korean pine stands in temperate zones. The biochemistry of photosynthesis can apparently tolerate temperatures below 0°C. The findings verified that soil temperatures, rather than foliar biochemistry, is more important to the onset of photosynthesis of pine trees.
